# PERSPECTIVEs on supervised exercise programs in people with metastatic breast cancer- a qualitative study in four European countries

**DOI:** 10.1007/s00520-023-07739-x

**Published:** 2023-04-19

**Authors:** Johanna Depenbusch, Maike G. Sweegers, Neil K. Aaronson, Yvonne Wengström, Malin Backman, Juan I. Arraras, Melanie Schranz, Britta Büchler, Milena Lachowicz, Anne M. May, Karen Steindorf, Martijn M. Stuiver

**Affiliations:** 1 Division of Physical Activity, Prevention and Cancer, German Cancer Research Center (DKFZ) and National Center for Tumor Diseases (NCT) Heidelberg, Heidelberg, Germany; 2 Center for Quality of Life, Netherlands Cancer Institute, Amsterdam, The Netherlands; 3 Division of Psychosocial Research and Epidemiology, Netherlands Cancer Institute/Antoni van Leeuwenhoek Hospital, Amsterdam, the Netherlands; 4 Division of Nursing, Department of Neurobiology, Care Sciences, and Society, Karolinska Institute and Karolinska Comprehensive Cancer Center, Karolinska University Hospital, Stockholm, Sweden; 5 Oncology Departments, Complejo Hospitalario de Navarra, Pamplona, Spain; 6 Institute of Medical Biostatistics, Epidemiology and Informatics (IMBEI), University Medical Center of the Johannes Gutenberg University Mainz, Mainz, Germany; 7 Department of Oncology and Radiotherapy, Medical University of Gdańsk, Gdańsk, Poland; 8 Julius Center for Health Sciences and Primary Care, University Medical Center Utrecht, Utrecht University, Utrecht, The Netherlands

**Keywords:** Metastatic breast cancer, Exercise, Barriers, Preferences, Focus groups, Qualitative research

## Abstract

**Purpose:**

Supervised exercise is a potentially promising supportive care intervention for people with metastatic breast cancer (MBC), but research on the patients’ perspective is limited. The aim of the current focus group study was to gain an in-depth understanding of MBC patients’ perceived barriers, facilitators, and preferences for supervised exercise programs.

**Methods:**

Eleven online focus groups with, in total, 44 MBC patients were conducted in four European countries (Germany, Poland, Spain, Sweden). Main topics of the semi-structured discussions covered attitudes towards participation in supervised exercise programs, perceived facilitators, experienced barriers, and exercise preferences. Interviews were transcribed verbatim, translated into English, and coded based on a preliminary coding framework, supplemented by themes emerging during the sessions. The codes were subsequently examined for interrelations and re-organized into overarching clusters.

**Results:**

Participants had positive attitudes towards exercise, but experienced physical limitations and insecurities that inhibited their participation. They expressed a strong desire for exercise tailored to their needs, and supervision by an exercise professional. Participants also highlighted the social nature of group training as an important facilitator. They had no clear preference for exercise type, but rather favored a mixture of different activities. Flexible training modules were considered helpful to increase exercise program adherence.

**Conclusions:**

MBC patients were generally interested in supervised exercise programs. They preferred group exercise that facilitates social interaction, but also expressed a need for individualized exercise programs. This suggests the relevance to develop flexible exercise programs that are adjusted to the individual’s needs, abilities, and preferences.

**Supplementary Information:**

The online version contains supplementary material available at 10.1007/s00520-023-07739-x.

## Introduction

Breast cancer is the most commonly diagnosed cancer in women worldwide [1]. Approximately 30% of affected women will develop metastatic disease. Although breast cancer remains the leading cause of female cancer-related death and the median 5-year survival rate for metastatic disease remains low (25%) [2], earlier detection and advances in oncological treatment have resulted in increasing survival rates [3, 4]. Hence, more patients are living with metastatic breast cancer (MBC) and concomitant symptoms of the disease and long-term treatment, such as fatigue, pain, or anxiety [5, 6]. As these symptoms contribute to a deterioration of patients’ quality of life (QoL), effective interventions are needed.

Exercise has been demonstrated to be a successful strategy for counteracting treatment-related side-effects and improving QoL among breast cancer patients receiving adjuvant treatment [7]. Recent studies have indicated that exercise might also play an important role in supportive care for breast cancer patients with advanced disease. The findings suggest that exercise is generally safe and feasible [8–11] and might positively affect patients’ physical fitness [9, 12, 13], fatigue, and QoL [9, 12].

Despite these promising findings, many MBC patients do not engage in exercise. Yee et al. compared physical activity (PA) levels between MBC patients and healthy controls and found significant differences regarding daily steps and weekly minutes of moderate-to-vigorous PA [14]. In a sample of mixed cancer types (~ 25% breast cancer), patients with metastatic disease reported significantly lower levels of aerobic and resistance exercise than cancer survivors [15]. The complex medical situation of MBC patients and their own, as well as health care providers’ (HCP) safety concerns may inhibit them from engaging in exercise [16–18], indicating the need for specific exercise programs.

To ensure exercise safety and effectiveness, instruction by a trained professional is advisable [19, 20], while the content of an exercise program can be designed according to patients’ perceived barriers, facilitators, and preferences to improve exercise adherence. Previous research on this topic has largely been limited to early-stage breast cancer patients [21, 22] or mixed cancer types [23–26], whose needs and preferences may differ from those of patients with MBC [5, 6].

The aim of the current focus group study was to identify facilitators, barriers, and preferences for supervised exercise programs among MBC patients in four European countries. As part of a larger project which aims to inform the implementation of exercise as supportive palliative care for MBC patients across Europe, country-specific differences were also explored. The qualitative approach was considered appropriate to better understand MBC patients’ perspectives in the context of their distinct cultural and disease-related situation [27].

## Methods

### Study design and participants

Our focus group study was conducted between 03/2021 and 05/2022 as part of a mixed methods, observational study (the PERSPECTIVE study) which is part of a larger EU-Horizon 2020 research program (the PREFERABLE project) investigating exercise interventions in the context of MBC [28]. Focus groups were held as online sessions via the platform Zoom (Zoom Video Communications, USA) in Poland, Spain, and Sweden, and via BigBlueButton in Germany, with 3–5 MBC patients per session. Participants were eligible if they: (1) had a histologically confirmed diagnosis of MBC; (2) were ≥ 18 years of age; (3) had an ECOG (Eastern Cooperative Oncology Group) performance status ≤ 2; and (4) had sufficient command of the local language. Patients were excluded if they had a life expectancy of < 6 months, were unable to perform basic activities of daily living, or showed severe cognitive problems. Both individuals with and without experience in supervised exercise programs were enrolled to identify factors influencing the potential uptake as well as the maintenance of exercise participation. Each study center used individual recruitment strategies and obtained ethical approval from their respective Ethics Committee (see Table 1 for more details). All participants signed informed consent.

**Table 1:** Information on moderators’ profession, recruitment strategies, and ethical approval per study center

Study center	*N*	Moderators/Observers	Profession	Methodological training	Recruitment strategies	Ethical approval
Mainz, DE	6	Melanie Schranz	Anthropologist, *PhD*	Research training in qualitative methods on a post-graduate level; additional courses in qualitative methods	Patients approached by treating physician; display of project information (poster); project announcement via mailing list of self-help groups	Ethik-Kommission bei der Landesärztekammer Rheinland-Pfalz
		Britta Büchler	Epidemiologist	Research training in qualitative methods on a post-graduate level; additional courses in qualitative methods		
Pamplona, ES	13	Juan I.Arrarás	Psychologist, *PhD*	Research training in qualitative methods on a doctoral level	Patients approached by treating physician	Comité Ético de Investigación Clínica de Navarra—Servicio Navarro de Salud
		Uxue Zarandona	Psychologist	Research training in qualitative methods on a post-graduate level		
Gdansk, PL	12	Paula Nyklewicz	Psychologist	Research training in qualitative methods on a post-graduate level	Former participants of the PREFERABLE project^a^ contacted again by study team (initially approached by treating physician)	Niezależna Komisja Bioetyczna do Spraw Badań Naukowych przy Gdańskim Uniwersytecie Medycznym
		Magdalena Mirek	Psychologist	Research training in qualitative methods on a post-graduate level		
Stockholm, SE	13	Yvonne Wengström	Nurse, *Professor*	Research training in qualitative interviewing on a doctoral level	Patients approached by treating physician or contact nurse for overall PREFERABLE project^a^ (incl. PERSPECTIVE focus group study); display of project information (leaflets) in waiting rooms	Etikprövningsmyndigheten
		Malin Backman	Nurse*, PhD*	Research training in qualitative interviewing on a doctoral level		

*DE* Germany; *ES* Spain; *PL* Poland; *SE* Sweden

^a^ EU-Horizon 2020 funded research program on exercise interventions in the context of MBC

### Data collection

To identify patients’ perspectives on supervised exercise programs, we used a semi-structured approach. Focus group moderators used an interview guide that contained broad and in-depth, open-ended questions on the three target topics, i.e., facilitators, barriers, and preferences for supervised exercise programs (see supplemental material). During the sessions, natural discussion flow and adequate coverage of relevant aspects were desired, while suggestions for the duration of the discussion of each topic in the interview guide facilitated time management. The interview guide was developed following the guidelines proposed by Moser and Korstjens [29]. First, topics of interest were collected from several health behavior models, including the social-cognitive theory [30] and the theory of planned behavior [31], and the investigators’ experiences from previous research [32–34]. After discussing and prioritizing the topics in the larger research team, an initial version of the interview guide was drafted under the supervision of two senior researchers (MMS; NKA) with extensive knowledge and experience in social sciences and exercise oncology. Feedback from the study group was obtained and implemented and the agreed upon version translated into all target languages.

At the beginning of each session, participants were asked to briefly introduce themselves and to describe their attitudes towards exercise, including whether this had changed after the diagnosis of MBC. The moderators then introduced the questions of the interview guide, also using prompts to encourage an interactive discussion. All moderators were experienced in leading focus group discussions and received study-specific training in the form of at least one briefing session to discuss the interview questions, the desired level of structure for the sessions, and the use of prompts. Moderators were supported by observers from their research team, who monitored the flow of the discussion and coverage of all targeted questions. After the first three focus group sessions, transcripts were preliminarily reviewed by two researchers (MGS; JD) and discussed in the larger research team. Moreover, moderators were asked about the practicality and usefulness of the interview guide. Given the shared impression that the discussions tended to drift off towards general PA as compared to supervised training, a few modifications were made to the interview guide to maintain the focus on supervised exercise programs.

Prior to the focus group sessions, participants completed a short online survey including items on sociodemographic characteristics, i.e., age, sex, home location (i.e., urban, suburban, rural), marital status, educational level, employment status, and body mass index. Self-reported medical information included locations of metastases, completed and ongoing cancer treatments, and co-morbidities. The Godin-Shephard Leisure-Time Physical Activity Questionnaire was used to assess exercise behavior in terms of weekly minutes of light, moderate, and vigorous-intensity aerobic activities and strength exercises in a typical week before the Coronavirus outbreak [35].

### Data analysis

All focus groups were audio- or videotaped, transcribed verbatim, translated into English, and imported into the software package MAXQDA Analytics Pro (Release 22.0.1). A deductive approach was used for thematic content analysis of the discussions [36, 37]. Two post-doctoral researchers (MGS, movement scientist with experience in qualitative research; JD, psychologist) independently coded all relevant statements using an unconstrained coding matrix that was defined by questions from the interview guide and supplemented by topics emerging during the sessions [36]. Coded statements were compared between the two investigators and, in case of mismatch, discussed until consensus was reached. As a means of investigator triangulation [38], the interpretation of ambiguous statements and a summary of each session were reviewed by the respective focus group moderators. The final agreed coding set was then analyzed for relationships between codes within and across the three major topics [37]. Rereading all coded statements allowed for the identification of common code combinations within single statements and the use of codes across different main topics. Based on these relationships, codes were restructured into overarching theme clusters, each of which was reviewed by all moderators and the larger research team.

## Results

### Patient characteristics

Eleven focus groups with a total of 44 MBC patients were conducted. Participants were all female with an average age of 53 years (*SD* = 9.2). The most frequent locations of metastases were bones (61.4%), liver (47.7%), and lungs (22.7%). About half of the participants received hormone therapy and 30% were currently undergoing chemotherapy. The majority of women reported having co-morbid conditions such as back pain (27.3%), high blood pressure (25.0%), or depression (15.9%). The median levels of self-reported PA were 102.5 min/week for light-intensity (interquartile range (*IQR*) = 45-120 min/week) and 120 min/week for moderate-intensity aerobic exercise (*IQR* = 36-160 min/week), whereas the median minutes for vigorous-intensity aerobic and resistance exercises were only slightly above zero (Table 2).

**Table 2:** Descriptive statistics of sample characteristics (*N* = 44)

	*N* or *M* or *Mdn*	% or *SD* or* IQR*
Age (*M*, *SD*)	53.0	9.2
Body mass index^a^		
Underweight	5	11.4
Normal weight	22	50.0
Overweight	13	29.5
Obese	4	9.1
Area of residence		
Urban	30	68.2
Suburban	9	20.5
Rural	4	9.1
Not sure	1	2.3
Marital status		
Married/living with a partner	34	77.3
Divorced/separated	6	13.6
Unmarried/single	4	9.1
Highest educational level^b^		
Academic education	27	61.4
Higher education	11	25.0
Middle education	4	9.1
No or basic education	2	4.5
Current employment status		
Employed	26	59.1
Not employed	18	40.9
Location of metastases^c^		
Bones	27	61.4
Liver	21	47.7
Lung	10	22.7
Brain	4	9.1
Other	14	31.8
Current treatment^c^	41	93.2
Chemotherapy	13	29.5
Radiotherapy	3	6.8
Hormone therapy	25	56.8
Immunotherapy or targeted therapy	20	45.5
Bone-modifying agents	12	27.3
Co-morbidities^c^		
Heart disease	4	9.1
Liver disease	6	13.6
High blood pressure	11	25.0
Diabetes mellitus	4	9.1
Back pain	12	27.3
Depression	7	15.9
Current physical activity (min/week)		
Light intensity aerobic exercise *(Mdn, IQR)*	102.5	45.0–120.0
Moderate intensity aerobic exercise *(Mdn, IQR)*	120.0	36.0–160.0
Vigorous intensity aerobic exercise *(Mdn, IQR)*	0.0	0.0–110.0
Strength/resistance exercise *(Mdn, IQR)*	2.5	0.0–112.5

*M* mean, *SD* standard deviation, *Mdn* Median, *IQR* interquartile range

^a^ Classification according to WHO: underweight: < 18.5 kg/m^2^; normal weight: 18.5–24.9 kg/m^2^; overweight 25–29.9 kg/m^2^; obese: > 30 kg/m^2^

^b^ Academic education: Bachelor degree or higher (according to Europe-wide Bologna process); higher education: degree qualifying for university; middle education: degree qualifying for further vocational training

^c^ Multiple answers possible

### Perspectives on supervised exercise programs

The content analysis of the focus group discussions resulted in five themes, which are detailed in the following sections: (1) MBC patients have a positive attitude towards exercising and expect multiple health benefits, (2) Physical barriers and insecurities require tailoring and supervision of exercise programs, (3) Social interactions and group training facilitate participation in exercise programs, (4) MBC patients have mixed preferences regarding exercise type and setting, (5) Exercise programming should allow flexibility in timing and intensity.

#### MBC patients have a positive attitude towards exercising and expect multiple health benefits

Participants generally expressed positive attitudes towards exercise. These were primarily instrumental in nature, i.e., focused on positive outcomes rather than emotions, and had not changed since the diagnosis of MBC. Despite more difficulties in pursuing exercise after the diagnosis, most women still regarded PA as important for their overall health and tried to incorporate exercise into their daily life.

‘Because of the cancer, I do it even more often. I try to do it every day. Unfortunately, endurance training in the form of jogging no longer works with my bones. I can't do that anymore. I have an indoor bike, a cross trainer and a rowing machine. I do yoga.’ (DE1_P2)^1^

Most women expected health benefits from exercise. Experiencing such improvements was an important reason for participating in exercise programs. Participants believed exercise would help maintain or improve their physical fitness, for example, in terms of increased lung capacity or regained strength, which in turn was expected to increase the tolerability of cancer treatment and facilitate physical functioning in everyday activities.

‘Physical fitness is also very important to get through a therapy well. So especially when you have another operation, or have this or that procedure; you know that the fitter I am, so to speak, the greater my ability to regenerate.’ (DE1_P3)

Moreover, exercise was regarded as beneficial for mental wellbeing in terms of increased energy levels and happiness as well as a feeling of calm and relaxation.

#### Physical barriers and insecurities require tailoring and supervision of exercise programs

Despite having positive attitudes, participants reported several factors that withheld them from joining supervised exercise programs. Physical limitations resulting from the disease or its treatment appeared as one of the main barriers. They seemed to not only directly hamper exercise, but to also cause insecurities regarding the optimal type and intensity. Some patients even reported a fear of harming themselves through exercise. Consequently, the large majority of women wanted to be supervised by a physiotherapist or an exercise professional. They believed that the professional could help them overcome their barriers by providing an individually tailored program and ensuring that exercises were carried out correctly.

‘Supervised exercises, i.e., under the supervision of a specialist, would certainly dispel any doubts that I would harm myself or do something that instead of having a positive effect on my body could cause harm, for example due to overloading the body.’ (PL3_P3)

In this context, some patients expressed a preference for exercising individually, rather than in a group. They felt that in one-on-one sessions, the exercise professional could better focus on their individual needs, provide encouragement, and give regular feedback on one’s progress.

#### Social interactions and group training facilitate participation in exercise programs

Although some patients favored exercising individually with a personal trainer, many women preferred group training as they enjoyed the opportunity to socialize during exercise. Group exercise was further seen to facilitate exercising regularly through commitment. Despite their agreement on benefits of group exercise, the participants expressed ambivalent opinions regarding group composition, both within- and between individuals. On the one hand, exercising in a group of women in a comparable situation was found to create a sense of belonging, the feeling of being understood, and the opportunity to share experiences. One participant described negative emotions and a feeling of not fitting in when attending “regular” exercise classes.

‘Because when I go to a fitness class, […] then I compare in any case me with others and then I realize, damn, how bad I am compared to many others. But when I meet people who have the same problems, who have had surgery etc., I can get support and we can also talk to each other about different problems and difficulties.’ (SE1_P4)

On the other hand, exercise was regarded as an occasion to shift the focus away from cancer, which led to a preference for a more heterogeneous exercise group.

‘It‘s true that sometimes you want to get together with people like you who have the same problems […], but sometimes what you want is just the opposite; to get together with people who have no disease and that the center of attention or conversation is not cancer.’ (ES1_P2)

#### MBC patients have mixed preferences regarding exercise type and setting

The discussions did not yield a clear shared preference for exercise type, but the participants were generally open to a mixture of different activities. For instance, aerobic and strengthening exercises were favored given the expected benefits of losing body weight and increasing muscle strength. Likewise, yoga and other mind–body exercises were mentioned in conjunction with improved mental wellbeing. Many women favored dancing classes, describing a feeling of joy when exercising with music and other people.

‘I like moving to music. Because that's what makes me happy. And then, if you get a positive feeling when you exercise to music, it makes you want to continue.’ (SE3_P2)

There was also no clear preference expressed by participants for a specific exercise setting. Instead, certain exercise types seemed to be associated with specific locations. Activities such as walking were preferred to be performed outdoors because of the opportunity to enjoy nature. For mind–body exercises or strength training, an exercise facility with appropriate equipment was considered to be most suitable and to facilitate exercising at higher intensities. However, some patients raised safety concerns regarding exercising in enclosed spaces with other people due to an increased risk of a Covid-19 infection.

‘I'd like to do that outside. To breathe as much oxygen as possible. I wouldn't like to stand on a running track in a gym. But then I would also like to go to a fitness center to specifically build up muscles.’ (DE1_P3)

Only one participant mentioned the hospital, but she would not prefer this as an exercise location because of the association with her disease. When explicitly asked about the hospital setting, some women shared this negative emotion, while others reacted neutral.

#### Exercise programming should allow flexibility in timing and intensity

Despite a perceived sense of accountability and commitment to a fixed exercise schedule, some participants highlighted that an exercise program should be flexible in terms of timing and intensity. Some women preferred supervised exercise programs that can be performed at flexible times given their work-related time restrictions or family responsibilities. Others described that their physical condition varied during their treatment cycles so that they would like to choose the time and intensity of exercise depending on how they felt.

‘One day you wake up very well, on the other you wake up feeling badly. That's why I think it's important to have the flexibility. […] If I'm feeling a bit tired, I decide to do Pilates that day. If I see that I'm rested and I'm fine, I go spinning.’ (ES2_P4)

An idea that was discussed in this context was the possibility of exercising at home, ideally supported by a digital program consisting of different exercise options. Nevertheless, participants emphasized that this should not replace, but rather supplement personal exercise counseling and supervised on-site training.

### Differences between participating countries

The discussions indicated only minor intercultural differences in MBC patients’ perspectives on supervised exercise. In particular, the Spanish women from the Navarra region emphasized the wish for social interaction in a group setting, while the benefits of one-on-one training were discussed more intensely among Swedish participants from the larger Stockholm area. Slight differences also emerged from the discussion about the ideal exercise setting, with the Spanish participants mostly preferring outdoor exercise and the Swedish and German women bringing up the idea of a complementary home-based digital program. Overall, participants’ perceived facilitators, barriers, and preferences for exercise seemed to differ more on an interpersonal than intercultural level.

## Discussion

This focus group study yielded insights regarding MBC patients’ perspectives on supervised exercise programs. Generally, participants expressed positive attitudes and outcome expectations towards PA despite their physical limitations due to MBC and its treatment. Support of and supervision by exercise professionals was regarded as crucial to overcome physical barriers and insecurities. Social contacts were cited as another important facilitator, which was further reflected by patients’ preference for group exercise. Participants did not have one shared preference regarding the exercise type, but named different forms of exercise, for which the setting should fit the activity. Finally, given women’s personal responsibilities, as well as treatment-related variation in physical abilities, flexible exercise programs consisting of different modules were considered as helpful to facilitate adherence to an exercise program.

Overall, we found that exercise facilitators, barriers, and preferences of MBC patients were comprised of cognitive, behavioral, and environmental factors that, as is posited in many health behavior models, do not act independently, but are correlated with one another [39]. Despite their advanced disease, participants regarded exercising as generally beneficial for their health and helpful in coping with their disease and treatment and expressed interest in a variety of exercise types and intensities, which is an encouraging finding. However, the possibility to exercise seemed to be limited by disease- and treatment-related physical constraints and resulting insecurities about the appropriate exercise type, amount, and execution. Insecurities and fear have been previously reported as exercise barriers in diverse cancer populations [21, 23, 40], but could be even more prominent for MBC patients given the adverse effects of their typically longer treatment history, having metastases and especially the (potential) presence of bone metastases. This could also explain women’s strong wish for professional supervision when exercising. Recent exercise recommendations indeed confirmed that patients with bone metastases should be supported by specifically trained exercise professionals, in consultation with the medical team [41]. This means that, to ensure the appropriateness of an exercise program, exercise professionals need to be educated on how to adjust an exercise program according to patients’ individual medical history [42]. Such professional reassurance could help to reduce patients’ insecurities and increase their sense of self-efficacy, which constitutes an important determinant of exercise maintenance [43].

Other factors perceived as exercise barriers or facilitators were comparable to those reported by early-stage breast cancer patients, for example the benefits of social interactions and patients’ overall preference for group exercise [21, 22, 44]. However, particular considerations for practical implementation must be made for this explicit target group, for instance regarding group composition. Many participants valued connecting with patients in a similar situation, which recommends making available exercise programs specifically designed for MBC patients. However, exercising in a group of patients with MBC might imply confrontation with adverse events such as progression of the disease or death of fellow patients. The handling of such sensitive topics must therefore be addressed when offering group exercise for patients with advanced disease. At the same time, some participants indicated a preference for “regular” exercise programs in order to get away from the feeling of being a patient. These individuals could, after undergoing a comprehensive medical assessment, be referred to more heterogeneous group classes that offer appropriate and safe exercises.

Different opinions also emerged around the issue of fixed appointments versus flexible exercise programs. While it is known that a fixed exercise schedule can be a helpful tool to stay focused and facilitate program attendance [17], particular focus should be given to MBC patients’ preference for program flexibility, as explained by their personal responsibilities and changeable physical condition. Offering additional exercise options such as digital home-based programs could increase exercise maintenance for these patients. Such programs should, however, be provided as complementary to on-site supervised exercise sessions. This recommendation reflects the view of breast cancer patients undergoing chemotherapy, who rated ‘hybrid’ programs with in-person and digital elements as most appropriate [40]. Yet, more research on the feasibility and effectiveness of such programs is needed, especially in the context of MBC.

To our knowledge, ours is the first study to carry out a qualitative analysis of European MBC patients’ views toward exercise programs. The focus group approach enabled us to gain a detailed understanding of the relationships between different factors influencing exercise participation in a so far understudied population. The results can be interpreted in light of the following strengths and limitations: First, participants’ relatively high PA levels and positive attitudes towards exercise suggest that our study sample may have been more ‘exercise-minded’ than the larger population of MBC patients. To develop a supervised exercise program that reaches all MBC patients, follow-up studies should recruit a more heterogeneous sample in terms of exercise-proneness. Second, although including individuals from four countries helped to broaden the geographic and cultural reach of our study, the reliability of cross-country comparisons is limited by the sample size as well as a restriction to particular regions within participating countries. Larger quantitative research studies are needed to validate potential intercultural differences. Further, the multicenter approach may have introduced some additional heterogeneity in methods such as different sampling strategies. Third, inherent to focus group discussions is the fact that moderators may have different interviewing skills and styles. However, the common interview guide, the interim evaluation of the first transcripts, and the investigator triangulation ensured that the most important questions regarding supervised exercise programs were addressed in all focus group discussions. Finally, there are remaining uncertainties that result from the scope of our study, and should therefore be addressed in future research. For instance, studies focusing on subgroups based on metastasis location or treatment type (chemotherapy, hormone therapy, etc.) could provide more precise information about supervised exercise programs needed for MBC patients. Moreover, there might be higher-level factors (e.g., regional differences regarding availability of exercise facilities, exercise counseling by HCPs, or health care insurance coverage) that need to be considered when designing and implementing exercise programs.

When implementing supervised exercise programs for MBC patients, a one-size-fits-all approach is not advised, given patients’ individual needs and preferences. A group exercise program targeting aerobic and strength capacities, and including mind–body exercises or possibly dancing seems a sensible approach but should be modified based on the individual’s health condition and personal circumstances. Supervision by exercise professionals is essential to adjust exercise according to patients’ capacity and help them overcome physical barriers and insecurities. Future research is needed to rigorously evaluate how to successfully implement such supervised exercise programs for patients with MBC.

## Supplementary Information

Below is the link to the electronic supplementary material.

Supplementary file1 (DOCX 17 KB)

## Data Availability

The authors declare that they have full control of all primary data and that they agree to allow the journal to review the data.

## Footnotes

1 Pseudonymized sources of verbatim quotations cite country of origin (DE: Germany; ES: Spain; PL: Poland; SE: Sweden), focus group, and interview participant.
