# Supplementary material for: PERSPECTIVEs on supervised exercise programs in people with metastatic breast cancer- a qualitative study in four European countries
Source: Support Care Cancer. 2023 Apr 19;31(5):281. doi: 10.1007/s00520-023-07739-x (PMC10115708; doi:10.1007/s00520-023-07739-x)
Supplement: Supplementary file 1 — Supplementary file1 (DOCX 17 KB) [file 520_2023_7739_MOESM1_ESM.docx]

Table S1. Main part of the interview guide containing questions on metastatic breast cancer patients’ attitudes, facilitators, barriers and preferences for supervised exercise programs.

| Topic | Wording | Time |
| --- | --- | --- |
| INTRODUCTION (QUESTION) | | |
|  | *Please tell us in a few words, what you think about exercise, in general, and whether your feelings about exercise have changed after having been diagnosed with metastatic breast cancer?* | maximum of about 5 minutes. |
| Suggestion for the moderator | When answers become lengthy, the moderator should guide the participants to shorten their answers for the introduction question. |  |
| KEY QUESTION 1 OUTCOME EXPECTATIONS | | maximum of 20 minutes |
| 1) Primary question | *If you were to engage in a supervised exercise program as described earlier, what would be the most important reason for you to participate?* |  |
| Suggestion for the moderator | If necessary, use prompts: e.g.,   - to improve your physical condition; - to build up stamina or reduce fatigue; - to improve your mood; for social contacts.   but avoid communicating the evidence with regard to such outcomes |  |
| Follow up question | *Could you explain why this reason is important to you?* |  |
| - 1. Sub question | *Are or would there be other reasons for you to participate in such an exercise program?* |  |
| - 1. Sub question | *What do you expect in terms of health benefits?* |  |
| Follow up question | *Could you explain whether this outcome is important to you and why?* |  |
| KEY QUESTION 2: BARRIERS | | maximum of 20 minutes |
| 2) Primary question | *Many people describe barriers to start and/or maintain exercising.*  *What barriers did you experience or do you think would keep you from joining a structured exercise program supervised by a physiotherapist or exercise professional as described earlier?* |  |
| Suggestions for the moderator | If necessary, use prompts: e.g.,  - practical barriers like costs or no training facilities close by;  - behavioral barriers like lack of motivation,  - concerns about safety issues.  - emotional barriers |  |
| Follow up question | In case participants mention barriers related to Covid-19:  *Would this barrier still be present once the pandemic is ended?* |  |
| 2.1) Sub question | For specific barriers that participants have mentioned:  *How did or could you overcome this barrier?* |  |
| 2.2) Sub question | *How could a physiotherapist or exercise professional assist or support you in overcoming such barriers?* |  |
| KEY QUESTION 3: PREFERENCES | | maximum of 20 minutes |
| 3) Primary question | *There are many different types of supervised exercise, such as aerobic or cardio classes, yoga or pilates, or resistance training working with weights. What type of exercise would you prefer in a supervised exercise program?* |  |
| 3.1) Sub question | *Could you further explain why this type of exercise is most appealing to you?* |  |
| Suggestions for the moderator | Please probe whether these preferences are based on prior experiences or rather on beliefs that people hold. |  |
| 3.2) Sub question | *What type and level of supervision from a physiotherapist or exercise professional would you prefer?* |  |
| 3.3) Sub question | *On which aspects of exercise or training would you like to receive advice from a physiotherapist or exercise professional*? |  |
| 4) Primary question | *Could you describe whether you prefer individual or group sessions and why?*  When group: *With whom would you like to exercise? For example with women with the same health problem, or would you prefer a mixed group of people?* |  |
| 5) Primary question | *Which setting (i.e., location of the exercise program) would you prefer and why?* |  |
| Suggestions for the moderator | If necessary, use prompts: e.g.,   - Indoor/outdoor - Gym - Hospital - At home |  |
| FINAL QUESTION | | Maximum of 5 minutes |
| 1) Primary question | *If you could develop an exercise program yourself, regardless of any obstacles such as time or finance, what would make an exercise program attractive for you?* |  |
| - 1. Sub question | We know that people sometimes have difficulty in following an exercise program over a longer period of time. What would it take to get you to participate in a supervised exercise program for a sustained period of time, or to have it as part of your lifestyle? |  |
